# Clinical Outcomes of the H-Shaped Incision in the Surgical Management of Naso-Orbito-Ethmoidal Fractures: A Prospective Clinical Study

**DOI:** 10.7759/cureus.117015

**Published:** 2026-09-27

**Authors:** Effie Edsor, Prabhusankar K, Srinisha M, Priyadharsana PS, Vignesh K, Vigneshwar S

**Affiliations:** 1 Department of Oral and Maxillofacial Surgery, Bharath Institute of Higher Education and Research, Chennai, IND; 2 Department of Oral and Maxillofacial Surgery, RVS Dental College and Hospital, Coimbatore, IND

**Keywords:** internal fixation, maxillofacial trauma, naso-orbito-ethmoidal fracture, open reduction, scar assessment

## Abstract

Background

Naso-orbito-ethmoidal fractures are complex central midfacial injuries that require adequate exposure for accurate reduction and stable fixation. This study evaluated the clinical outcomes of an H-shaped incision approach for naso-orbito-ethmoidal fractures.

Materials and methods

This prospective single-arm study included 20 adults with clinically and radiographically confirmed naso-orbito-ethmoidal fractures requiring open reduction and internal fixation. The central fracture region was exposed through an H-shaped incision with additional infraorbital or intraoral vestibular access when required. Outcomes included operative time, surgical exposure, fracture reduction, pain, facial edema, wound healing, scar quality, neurosensory status, and complications. Patients were followed for six months.

Results

The mean operative time was 85.15 ± 7.05 minutes. Surgical exposure was excellent in 12 (60%) patients and good in 8 (40%) patients. Fracture reduction was excellent in 13 (65%) patients and good in 7 (35%) patients. Mean pain scores decreased from 5.25 ± 0.85 at one week to 1.45 ± 0.51 at one month (p<0.001). Mean Patient and Observer Scar Assessment Scale scores decreased from 21.95 ± 2.35 at one month to 10.15 ± 1.50 at six months (p<0.001). Three (15%) patients developed transient paresthesia. No persistent sensory deficit or clinically apparent medial canthal tendon or nasolacrimal duct injury was recorded.

Conclusions

The H-shaped incision approach provided adequate surgical exposure and satisfactory fracture reduction with favorable postoperative recovery in selected patients. Larger comparative studies are required to define its relative advantages over established approaches.

## Introduction

Naso-orbito-ethmoidal (NOE) fractures are uncommon but clinically important injuries of the central midface. They involve the bony framework surrounding the nasal root, medial orbit, and ethmoid region, as well as structures related to the medial canthal tendon (MCT) and lacrimal drainage system. Disruption of these structures can alter intercanthal position, orbital support, and nasal projection. Inadequate reduction or stabilization may therefore result in persistent deformities such as traumatic telecanthus, enophthalmos, diplopia, epiphora, nasal deformity, and facial asymmetry [1,2]. Surgical treatment aims to restore the facial buttresses, orbital anatomy, nasal projection, and MCT position through accurate anatomical reduction and stable fixation [1,2].

Achieving these objectives requires adequate visualization of the fracture while limiting unnecessary soft tissue disruption. Recent systematic reviews have shown that open reduction and internal fixation is the most frequently reported treatment for displaced NOE fractures and that the coronal incision remains the most commonly described approach [1,3]. Although the coronal approach provides broad exposure for extensive injuries, it requires relatively wide dissection. Several local approaches have therefore been described for selected fracture patterns including existing lacerations, glabellar and vertical incisions, the Lynch approach, transcaruncular approaches, the butterfly incision, and the H-shaped incision [3,4]. Selection of the surgical approach depends on the fracture pattern, associated injuries, required exposure, and surgeon experience [3,4].

The H-shaped incision is a local cutaneous approach described for direct access to the NOE region [4]. Its relatively localized access may be useful in selected fractures. However, current evidence does not establish its superiority over other approaches with respect to surgical exposure, operative time, postoperative morbidity, or scar outcomes. Contemporary systematic reviews have also highlighted the limited quality of evidence supporting individual NOE repair techniques and the need for more robust prospective studies [1,3]. Therefore, this prospective clinical study evaluated the clinical performance and postoperative outcomes of the H-shaped incision in patients undergoing open reduction and internal fixation of NOE fractures. The evaluation included surgical exposure, fracture reduction and fixation, operative time, postoperative pain and edema, wound healing, scar quality, neurosensory function, and procedure-related complications during six months of follow-up.

## Materials and methods

Study design and participants

This prospective single-arm clinical study included 20 patients with NOE fractures who underwent surgical management in the Department of Oral and Maxillofacial Surgery, RVS Dental College and Hospital, Coimbatore, Tamil Nadu, India, between October 2025 and March 2026. All patients were followed prospectively for six months after surgery. The study protocol was reviewed and approved by the Institutional Review Board of RVS Dental College and Hospital (approval number: 129/ETHICS/2025). Written informed consent was obtained from all participants before enrollment. Eligibility was determined according to predefined clinical and radiographic criteria, which are summarized in Table 1.

**Table 1:** Inclusion and exclusion criteria for study participation

Eligibility category	Criterion
Inclusion	Age 18 years or older
Inclusion	Clinically and radiographically confirmed naso-orbito-ethmoidal fracture
Inclusion	Fracture requiring open reduction and internal fixation
Exclusion	Pathological fracture
Exclusion	Previous surgery involving the naso-orbito-ethmoidal region
Exclusion	Associated frontal bone fracture
Exclusion	Severe systemic illness that precluded surgery
Exclusion	Unwillingness to participate
Exclusion	Inability to complete the scheduled follow-up

Eligible patients with NOE fractures presenting during the predefined study period (October 2025 to March 2026) were enrolled consecutively. A formal a priori sample size calculation was not performed because this was an exploratory prospective single-arm clinical study. All eligible patients during the study period were included, resulting in a final cohort of 20 patients.

Preoperative assessment

All patients underwent detailed clinical and radiographic evaluation before surgery. Clinical examination included assessment of facial symmetry, nasal and orbital contour, intercanthal relationship, ocular function, facial sensation, and associated maxillofacial injuries.

Computed tomography (CT) of the facial skeleton with three-dimensional reconstruction was performed to determine the location, extent, and displacement of the NOE fracture and to identify associated facial fractures. All patients also underwent ophthalmologic evaluation before surgical intervention.

NOE fractures were classified using the Markowitz and Manson classification according to the relationship between the MCT and the central fracture fragment [5]. Type I fractures were characterized by an intact MCT attached to a relatively large central bony fragment. Type II fractures involved comminution of the central fragment with the MCT remaining attached to a bony fragment. Type III fractures involved severe comminution affecting the MCT-bearing central fragment with loss of stable bony attachment of the MCT. The extent of surgical exposure and fixation was planned according to the clinical findings, fracture classification, and three-dimensional CT assessment.

Surgical anatomy and H-shaped incision design

The NOE region includes the nasal bones, frontal process of the maxilla, lacrimal bone, ethmoid complex, and medial orbital wall. The MCT is particularly important because its attachment to the central bony fragment contributes to medial canthal position and stability. The lacrimal sac and nasolacrimal drainage pathway are also closely related to the operative field. Careful preservation of these structures is important during fracture exposure, reduction, and fixation [4,6]. The H-shaped incision was centered over the nasal region and consisted of two vertical limbs connected by a transverse horizontal limb [4]. The vertical limbs were positioned along the lateral nasal region in relation to the nasomaxillary buttresses. Superiorly, the vertical limbs terminated below the medial ends of the eyebrows. The transverse limb extended across the nasal dorsum and connected the two vertical limbs to form the H-shaped configuration (Figure 1).

**Figure 1:**
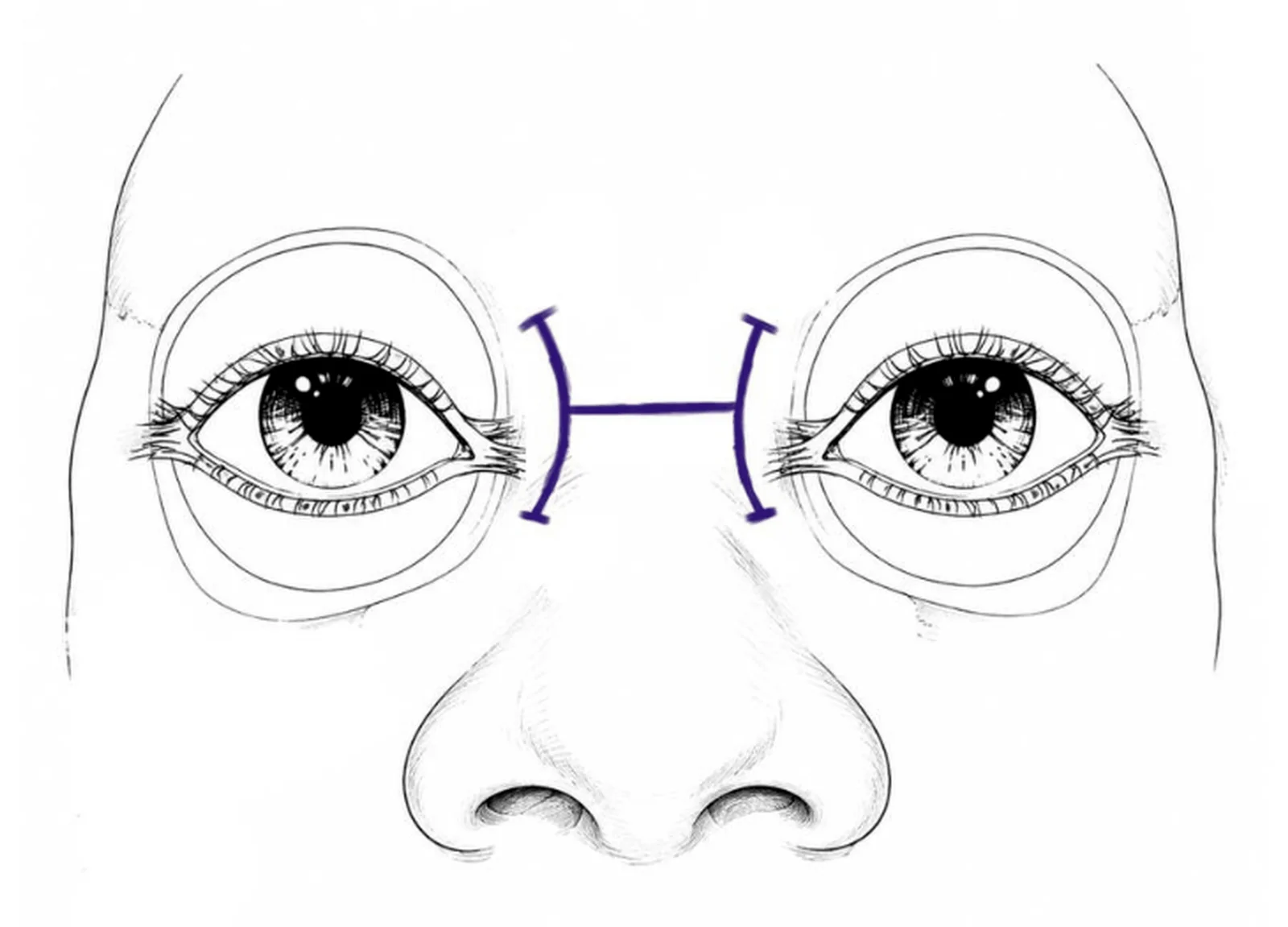
Schematic representation of the H-shaped incision used for surgical exposure of the naso-orbito-ethmoidal region The illustration was created by the authors using Inkscape.

Surgical procedure

All procedures were performed by the same oral and maxillofacial surgeon using a standardized surgical protocol under general anesthesia. Operative time was measured from placement of the first incision to completion of wound closure.

The central NOE complex was exposed through the H-shaped incision. Additional surgical access was used according to the location and extent of the fracture. An infraorbital incision was performed when additional exposure of the infraorbital rim was required, whereas an intraoral high vestibular incision was considered when exposure of the maxillary buttress was necessary. The H-shaped incision was used as the primary approach in all patients. Supplementary infraorbital access was required in two patients for additional exposure of the infraorbital region, while no patient required supplementary intraoral vestibular access.

Following adequate exposure, the fractured segments were identified and mobilized where required. Reduction was performed to restore the central midfacial framework, nasal contour, and naso-orbital relationship. Particular attention was given to the position and stability of the MCT-bearing central fragment.

Stable fixation was achieved using titanium miniplates and screws supplied by SK Surgicals, Pune, Maharashtra, India. The position, alignment, and stability of the reduced fracture segments were evaluated before wound closure. Medial canthopexy was performed in one patient when additional stabilization of the medial canthal region was required.

Layered wound closure was performed using 3-0 and 4-0 Vicryl sutures. Standard postoperative instructions included head elevation (even during sleep), intermittent cold compresses during the first 24-48 hours without direct pressure over the operative site, and maintenance of a clean and dry incision site. Patients were instructed to avoid nose blowing, forceful sneezing, heavy lifting, bending, strenuous physical activity, and manipulation or pressure over the nasal and orbital regions. When sneezing was unavoidable, patients were instructed to sneeze with the mouth open. A soft diet was advised during the early postoperative period. Figure 2 demonstrates the H-shaped incision used for exposure of the fracture site, and Figure 3 demonstrates open reduction and internal fixation of an NOE fracture using titanium miniplates and screws.

**Figure 2:**
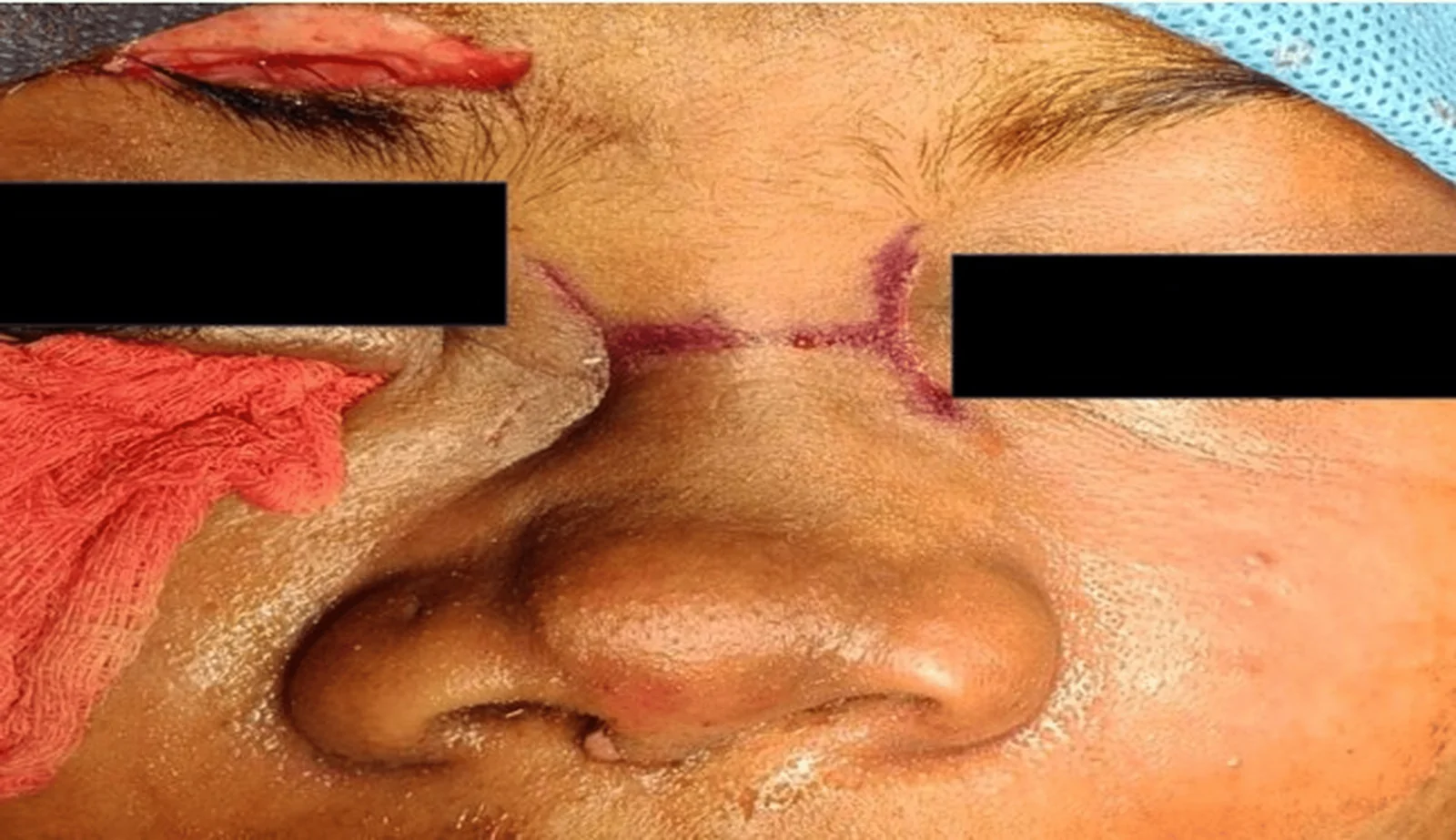
H-shaped incision used for exposure of the naso-orbito-ethmoidal fracture

**Figure 3:**
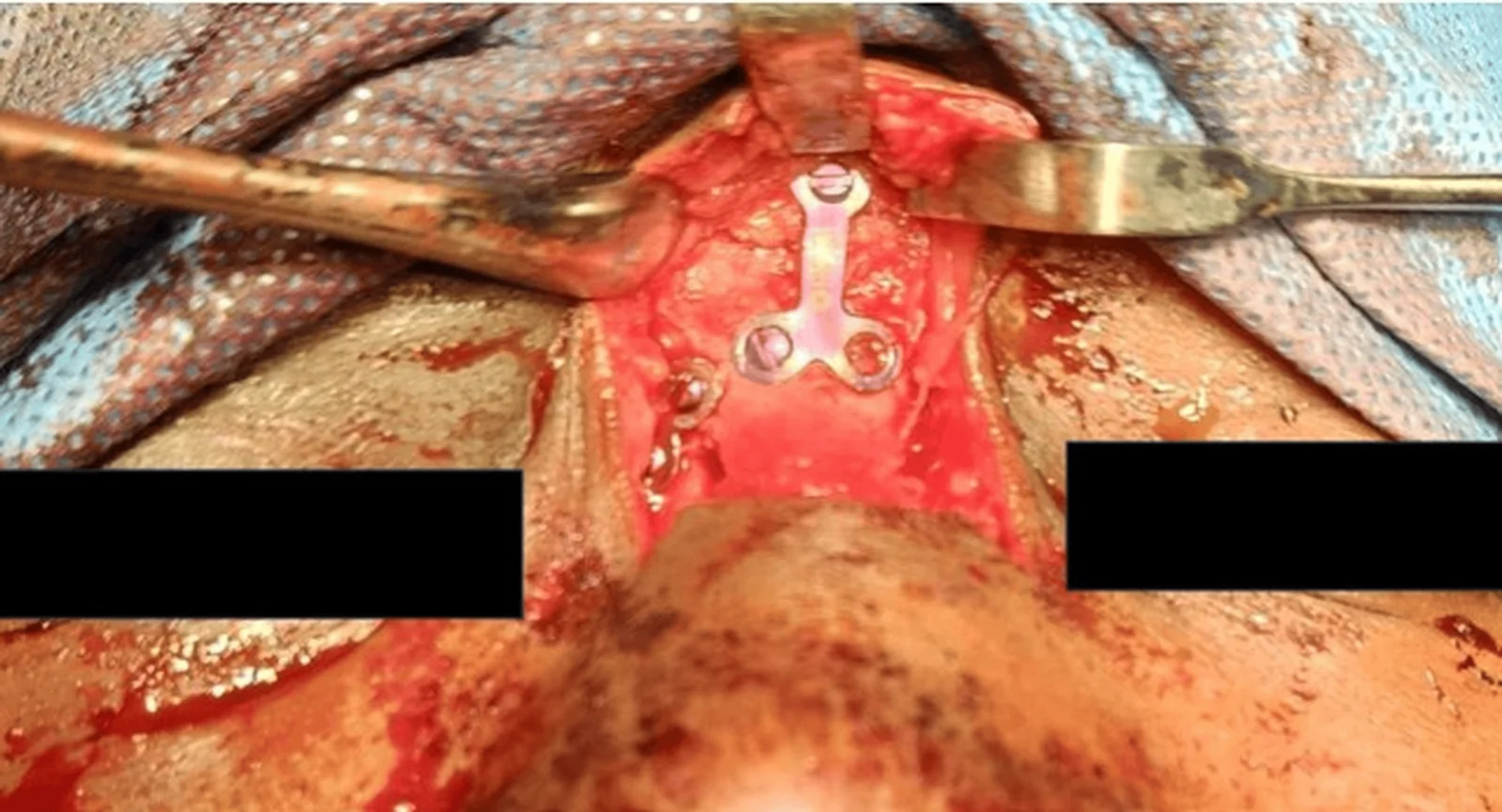
Open reduction and internal fixation of the naso-orbito-ethmoidal fracture using titanium miniplates and screws

Intraoperative outcome assessment

The intraoperative outcomes were operative time, adequacy of surgical exposure, and ease of fracture reduction and fixation.

Surgical exposure was assessed by the operating surgeon using an investigator-developed five-point ordinal scale created specifically for this study. This scale has not undergone formal external validation. A score of 1 indicated very poor exposure with inadequate visualization of the fracture site and major difficulty in reduction or fixation. A score of 2 indicated poor exposure with visualization of only part of the fracture site and considerable difficulty in reduction or fixation. A score of 3 indicated fair exposure with adequate visualization but moderate difficulty during reduction or fixation. A score of 4 indicated good exposure with clear visualization and reduction and fixation performed with minimal difficulty. A score of 5 indicated excellent exposure with wide and clear visualization of the relevant fracture site and easy reduction and fixation.

For reporting, the observed surgical exposure ratings were summarized according to these predefined ordinal categories.

Assessment of fracture reduction

The adequacy of fracture reduction was evaluated on postoperative CT using the radiographic criteria described by Lee et al. [7]. Postoperative CT assessment was performed independently by another surgeon who was not involved in the surgical procedure and by a radiologist. The assessors were blinded to the intraoperative surgical exposure assessment. Assessment was based on bony contact, deviation angle, and restoration of the nasal bony arch contour.

Reduction was categorized as excellent when bony contact was 75%-100%, the deviation angle was 0°-20°, and a smooth nasal bony arch contour was restored. Reduction was categorized as good when bony contact was 25%-75%, the deviation angle was 0°-20°, and the nasal bony arch contour was nearly normal. Fair reduction represented less than 25% bony contact with a deviation angle greater than 20° and an irregular nasal bony arch contour. Poor reduction represented undercorrection with persistent displacement irrespective of deviation angle or arch configuration.

The scale was originally described for radiographic assessment following nasal bone reduction and was applied in the present study to postoperative CT assessment of reduction in the central NOE region.

Postoperative pain assessment

Postoperative pain was assessed using a 0-10 visual analog scale (VAS) at one week and one month after surgery. A score of 0 represented no pain. Scores of 1-3 represented mild pain, scores of 4-6 represented moderate pain, scores of 7-9 represented severe pain, and a score of 10 represented the worst pain imaginable. Higher scores therefore represented greater pain intensity.

Facial edema assessment

Facial edema was evaluated clinically at one week and one month after surgery using a predefined five-grade ordinal scale.

Grade 0 indicated no edema. Grade 1 represented mild edema defined as minimal localized swelling without significant alteration of the facial contour. Grade 2 represented moderate edema characterized by obvious swelling with mild alteration of the facial contour. Grade 3 represented severe edema with marked diffuse swelling and significant alteration of the facial contour and/or periorbital swelling. Grade 4 represented very severe edema characterized by extensive tense swelling causing major facial distortion and/or severe periorbital edema.

Changes in edema grade during follow-up were used to characterize resolution of postoperative swelling.

Wound healing assessment

Wound healing was evaluated clinically during the first postoperative week. Healing was categorized as excellent when the incision demonstrated satisfactory approximation without clinically evident wound-related complications. Healing was categorized as good when satisfactory healing was present with minor local findings that did not require additional surgical intervention.

Scar assessment

Scar quality was assessed at one, three, and six months using version 2.0 of the Patient and Observer Scar Assessment Scale (POSAS) [8]. POSAS 2.0 incorporates complementary assessments from the patient and the clinical observer and evaluates multiple visual, tactile, and sensory characteristics of the scar. Both Patient Scar Assessment Scale and Observer Scar Assessment Scale components were completed during scar assessment. The final POSAS score used for analysis was calculated as the average of the Patient and Observer component scores. Higher scores represented greater deviation from normal skin, whereas lower scores indicated more favorable scar characteristics. Serial scores were used to evaluate scar evolution during the six-month follow-up period.

Neurosensory assessment

Infraorbital sensory function was evaluated using two-point discrimination testing during postoperative follow-up. The test was used to assess the patient's ability to distinguish two simultaneously applied points within the infraorbital sensory distribution.

Sensory disturbance was recorded as absent, transient, or persistent. Transient paresthesia was defined as altered postoperative sensation that resolved during the six-month follow-up period. Persistent sensory deficit was defined as altered sensation that remained at the six-month assessment.

Assessment of procedure-related complications

Patients were evaluated for procedure-related complications throughout follow-up. Particular attention was given to the MCT, nasolacrimal apparatus, infraorbital sensory function, and surgical wound.

The presence of medial canthal instability, clinically apparent nasolacrimal dysfunction, persistent paresthesia, wound-related complications, and requirement for additional surgical intervention was recorded. The integrity of the medial canthal region and nasolacrimal apparatus was assessed clinically during postoperative review.

Follow-up

Patients were evaluated at one week, one month, three months, and six months after surgery. The one-week assessment included pain, facial edema, wound healing, neurosensory status, and early postoperative complications. At one month, pain, residual edema, scar characteristics, sensory recovery, and procedure-related complications were evaluated.

Scar quality, neurosensory status, and procedure-related complications were further assessed at three and six months. Clinical and radiographic findings were reviewed during follow-up to evaluate maintenance of fracture reduction and fixation.

Statistical analysis

Statistical analyses were performed using IBM SPSS Statistics for Windows, Version 26.0 (IBM Corp., Armonk, NY, USA). Continuous variables were summarized using the mean and standard deviation. Categorical and ordinal variables were summarized using frequencies and percentages.

Changes in VAS pain scores between one week and one month were evaluated using the Wilcoxon signed-rank test because the measurements represented paired observations from the same participants. Changes in POSAS scores across one, three, and six months were evaluated using the Friedman test for repeated within-participant measurements. Pairwise comparisons were performed following a statistically significant overall Friedman test with adjustment for multiple comparisons.

Operative time was summarized using the mean and standard deviation. Surgical exposure, fracture reduction, facial edema, wound healing, neurosensory outcomes, and postoperative complications were summarized using frequencies and percentages. All statistical tests were two-sided, and a p-value of <0.05 was considered statistically significant.

## Results

All 20 patients completed the scheduled six-month follow-up period, and no patients were lost to follow-up. No missing outcome data were recorded at any predefined assessment time point. Baseline demographic and clinical characteristics of the study participants are summarized in Table 2. The study included 20 patients with NOE fractures, with an age range of 18-35 years. Thirteen (65%) patients were male, and 7 (35%) patients were female. According to the Markowitz and Manson classification, nine (45%) patients had type I fractures and 11 (55%) patients had type II fractures, while no type III fractures were observed. Associated injuries included mandibular condyle fractures in three patients (15%) and frontozygomatic fractures in five patients (25%) (Table 2).

**Table 2:** Baseline demographic and clinical characteristics of study participants

Characteristic	Category	Range	N (%)
Age (years)	-	18-35 years	-
Sex	Male	-	13 (65)
	Female	-	7 (35)
Markowitz and Manson classification	Type I	-	9 (45)
	Type II	-	11 (55)
	Type III	-	0 (0)
Associated injuries	Mandibular condyle fracture	-	3 (15)
	Frontozygomatic fracture	-	5 (25)

Intraoperative outcomes

The analysis included 20 patients with NOE fractures. The mean operative time was 85.15 ± 7.05 minutes. Surgical exposure was rated as excellent in 12 (60%) patients and good in 8 (40%) patients. Eighteen patients were managed using the H-shaped incision alone, while two patients required supplementary infraorbital access. No patient required supplementary intraoral vestibular access. No procedure required conversion to a more extensive surgical approach.

Fracture reduction was rated as excellent in 13 (65%) patients and good in 7 (35%) patients. Postoperative clinical and radiographic assessment demonstrated maintenance of fracture alignment and fixation. Representative preoperative and postoperative three-dimensional computed tomography images are presented in Figure 4.

**Figure 4:**
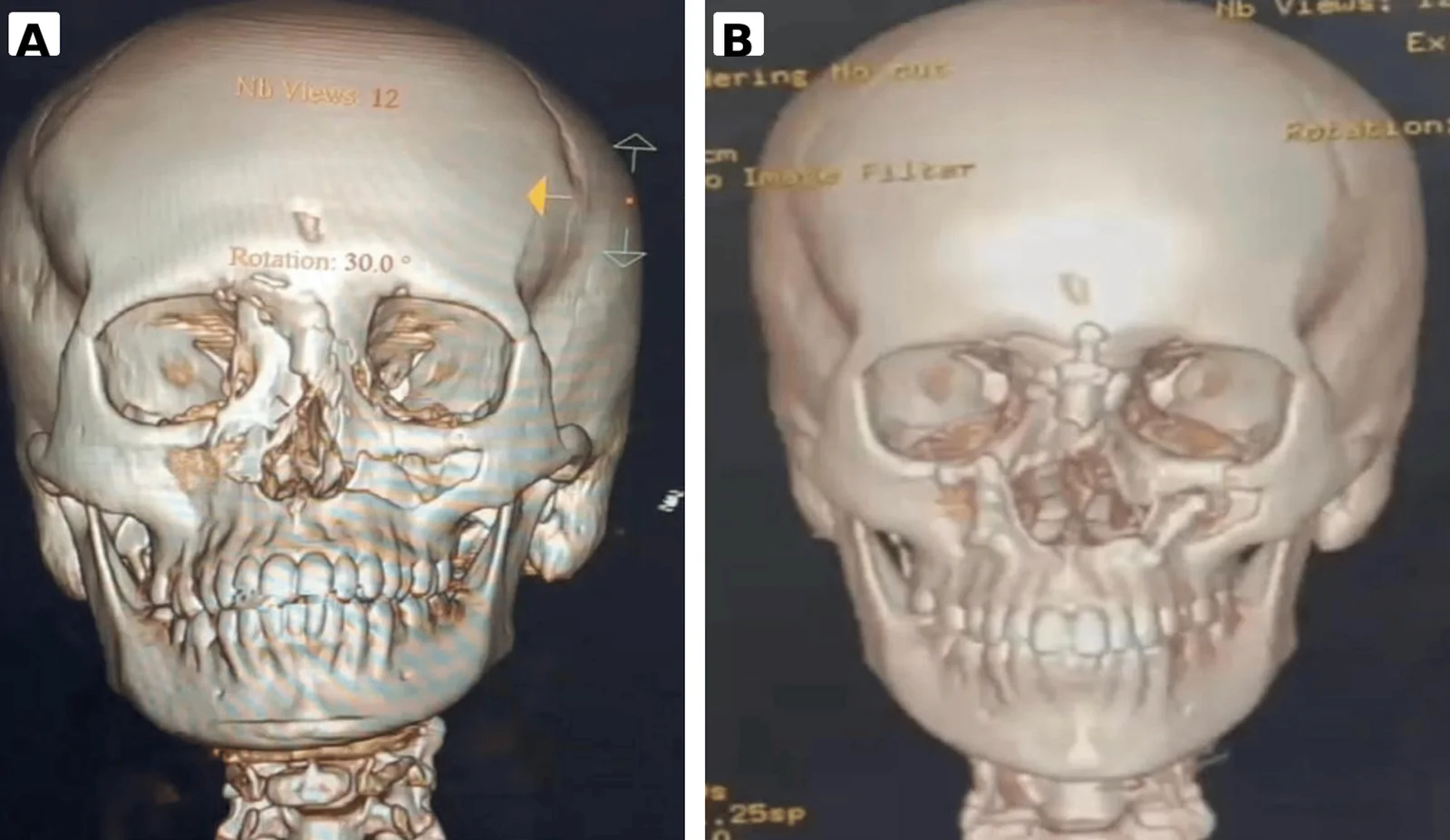
Preoperative and postoperative three-dimensional reconstructed computed tomography images of a patient with a naso-orbito-ethmoidal fracture

Postoperative edema and wound healing

At one week, facial edema was mild in 10 (50%) patients, moderate in 9 (45%) patients, and severe in 1 (5%) patient. No patient was free of edema at this assessment. At one month, edema had resolved completely in 13 (65%) patients, while 7 (35%) patients had mild residual edema. No moderate or severe edema was recorded at one month.

At the first postoperative week, wound healing was rated as excellent in 11 (55%) patients and good in 9 (45%) patients. No wound-related complication requiring additional surgical intervention was reported.

Postoperative pain

The mean VAS pain score decreased from 5.25 ± 0.85 at one week to 1.45 ± 0.51 at one month. The mean absolute reduction was 3.80 points. The paired reduction in pain was statistically significant (p<0.001) (Table 3). Mean reduction from one week to one month: 3.80 points; p<0.001.

**Table 3:** Postoperative pain assessed using VAS Mean reduction from one week to one month: 3.80 points; p<0.001. VAS, visual analog scale

Postoperative assessment	Mean VAS score	SD
One week	5.25	0.85
One month	1.45	0.51

Scar outcomes

The mean POSAS score demonstrated a progressive reduction during the six-month follow-up period. The mean POSAS score decreased from 21.95 ± 2.35 at one month to 15.15 ± 1.63 at three months and further decreased to 10.15 ± 1.50 at six months (Table 4).

**Table 4:** Mean POSAS scores during postoperative follow-up Overall comparison across one, three, and six months: p<0.001. POSAS, Patient and Observer Scar Assessment Scale

Postoperative assessment / comparison	Mean POSAS score ± SD / test statistic	p-Value
One month	21.95 ± 2.35	-
Three months	15.15 ± 1.63	-
Six months	10.15 ± 1.50	-
Overall comparison (Friedman test)	χ²(2) = 40.00	<0.001
Pairwise comparison: one month vs three months (Wilcoxon signed-rank test)	W = 0.00; Z = −3.981	<0.001
Pairwise comparison: one month vs six months (Wilcoxon signed-rank test)	W = 0.00; Z = −3.968	<0.001
Pairwise comparison: three months vs six months (Wilcoxon signed-rank test)	W = 0.00; Z = −4.300	<0.001

The Friedman test demonstrated a statistically significant difference in POSAS scores across the three postoperative assessment periods (χ²(2)=40.00, p<0.001). Post-hoc Wilcoxon signed-rank comparisons demonstrated significant reductions in POSAS scores between one month and three months (W=0.00, Z=−3.981, p<0.001), one month and six months (W=0.00, Z=−3.968, p<0.001), and three months and six months (W=0.00, Z=−4.300, p<0.001).

These findings indicate progressive improvement in scar characteristics throughout the six-month postoperative period, with continued scar maturation observed between each follow-up assessment.

Neurosensory outcomes and complications

No postoperative sensory disturbance was reported in 17 (85%) patients. Three patients (15%) experienced transient paresthesia during the first postoperative month. These sensory disturbances resolved during follow-up, and no persistent sensory deficit was recorded at six months.

No postoperative injury involving the MCT or nasolacrimal duct was recorded during the six-month follow-up period.

## Discussion

NOE fractures are among the more challenging injuries of the central midface because reconstruction involves the nasal framework, medial orbit, MCT, and lacrimal drainage system [1-3]. Successful treatment requires adequate visualization of the fracture segments with restoration of the central facial contour and stable fixation while minimizing additional soft tissue injury. In the present prospective study, the H-shaped incision approach provided excellent or good surgical exposure in all 20 patients. Fracture reduction was also rated as excellent or good in all cases. Postoperative pain and edema decreased during follow-up while scar scores improved progressively. Transient paresthesia occurred in three patients, but no persistent sensory deficit or clinically apparent injury to the MCT or nasolacrimal duct was recorded at six months.

Adequate surgical exposure is fundamental to NOE fracture management. Recent systematic reviews have shown that open reduction and internal fixation remains the predominant treatment for displaced NOE fractures and that the coronal approach continues to be widely used as it provides broad visualization of the upper and central facial skeleton [1-3]. However, the extent of surgical exposure required varies according to the fracture pattern, degree of comminution, and associated orbital or midfacial injuries. Local approaches may provide sufficient access in appropriately selected injuries and can be combined with additional incisions when adjacent facial buttresses require exposure [3,4]. In the present study, the H-shaped incision provided central access to the NOE region, while supplementary infraorbital or intraoral vestibular incisions were used when required. The findings therefore represent an H-shaped incision strategy rather than isolated use of the H-shaped incision for every component of fracture management.

The mean operative time was 85.15 ± 7.05 minutes. This provides a practical estimate of operative duration for the patients included in this series. Direct comparison with previously reported operating times is difficult because NOE fracture severity, associated injuries, number of fixation sites, and surgical access vary considerably between studies. Institutional experience with NOE reconstruction also demonstrates that operative planning is determined by the extent and complexity of the injury rather than by a single standardized approach [9]. The operative duration observed in the present study therefore cannot be interpreted as evidence that the H-shaped incision reduces surgical time relative to a coronal or other approach. A controlled comparison involving similar fracture patterns would be required to establish such a difference.

Fracture reduction was rated as excellent in 65% of patients and good in the remaining 35%. These findings indicate that satisfactory reduction and fixation were achievable with the surgical access used in this cohort. Open reduction and internal fixation is the most frequently reported treatment for displaced adult NOE fractures, and restoration of the central facial framework remains a principal objective of reconstruction [1,2]. The quality of reduction in the present study is therefore clinically relevant. However, the reduction categories were study-specific clinical assessments rather than measurements derived from a validated NOE reduction scale. In addition, the absence of a comparison group prevents attribution of the observed reduction quality exclusively to the H-shaped incision.

Preservation of the medial canthal region is particularly important because displacement or disruption of the MCT-bearing central fragment can result in persistent telecanthus and alteration of central facial appearance [4,5]. The nasolacrimal system is also vulnerable because of its close anatomical relationship to the NOE complex. Published evidence identifies telecanthus, postoperative epiphora, and nasolacrimal dysfunction among the clinically relevant sequelae of NOE fractures and their treatment [2,5]. No clinically apparent MCT injury or nasolacrimal duct injury was recorded in the present cohort during six months of follow-up. This represents a favorable finding within the study population. The small sample and clinical method of assessment limit broader conclusions regarding the risk of these complications.

Postoperative pain showed a marked reduction during early recovery. The mean VAS score decreased from 5.25 ± 0.85 at one week to 1.45 ± 0.51 at one month. Facial edema followed a similar clinical pattern. At one week, 50% of patients had mild edema, 45% had moderate edema, and 5% had severe edema. By one month, 65% had no residual edema, and the remaining 35% had only mild swelling. These changes are consistent with progressive postoperative resolution of soft tissue inflammation. Pain and edema after maxillofacial surgery are influenced by several factors including the severity of the primary injury, extent of tissue manipulation, duration of surgery, and additional surgical approaches. The improvement observed in this study therefore reflects postoperative recovery after the overall surgical procedure and cannot be attributed solely to the configuration of the H-shaped incision.

Early wound healing was also favorable. At the first postoperative week, healing was rated as excellent in 55% of patients and good in 45%. No wound-related complication requiring additional surgical treatment was reported. Limited local exposure may reduce the extent of tissue dissection in selected cases, but the present study was not designed to compare wound healing between incision types. Consequently, these findings demonstrate satisfactory healing within the cohort rather than a healing advantage over alternative surgical approaches.

Scar quality is particularly relevant when an incision is placed within the central facial region. POSAS provides a structured method for assessing scar characteristics from both patient and observer perspectives and has demonstrated reliability for clinical scar assessment [8,10]. In the present study, the mean POSAS score decreased from 21.95 ± 2.35 at one month to 15.15 ± 1.63 at three months and 10.15 ± 1.50 at six months. The progressive decline indicates improvement in recorded scar characteristics over time. The observed reduction in POSAS scores suggests favorable scar maturation during the six-month follow-up period. However, scar remodeling is a natural biological process, and in the absence of a comparative incision group, the improvement cannot be attributed solely to the H-shaped incision design.

The favorable scar evolution is clinically encouraging but requires careful interpretation. Scar appearance naturally changes during maturation, and improvement over several months is expected as remodeling progresses. Incision orientation and mechanical tension across a healing wound may also influence scar development. However, the proposed relationship between incision planning, skin tension, and hypertrophic scar formation remains incompletely established in clinical studies [11]. The observed POSAS improvement therefore cannot be attributed solely to the H-shaped design or its orientation. A comparative study using standardized scar assessment at identical follow-up intervals would provide stronger evidence regarding the aesthetic performance of different approaches.

Neurosensory outcomes were also favorable. Seventeen patients had no postoperative sensory disturbance, while three developed transient paresthesia during the first postoperative month. All transient disturbances resolved during follow-up, and no persistent deficit was present at six months. These findings indicate satisfactory clinical sensory recovery in this series. They remain limited by the absence of standardized quantitative neurosensory testing. Formal sensory testing could provide greater precision in future studies by distinguishing subtle changes in touch, pressure, and discrimination that may not be evident during routine clinical assessment.

An important feature of the present study is the use of the H-shaped incision as part of a flexible surgical strategy rather than as an isolated approach. Additional infraorbital and intraoral vestibular access was used when required by the fracture pattern. The requirement for supplementary approaches represents a potential confounding factor. In patients requiring additional infraorbital access, operative time, surgical exposure assessment, and postoperative outcomes may reflect the combined surgical approach rather than the isolated effect of the H-shaped incision. This reflects the clinical reality of NOE reconstruction in which the required exposure depends on the anatomical extent of injury [1,3,4]. The H-shaped incision appears useful for direct access to the central NOE region in selected patients. Extensive injuries involving the frontal region, wider orbital framework, or multiple facial buttresses may require broader exposure. The current findings therefore support feasibility in selected cases rather than replacement of established approaches.

Established principles of NOE fracture management emphasize detailed CT assessment, identification of the MCT-bearing central fragment, and selection of surgical exposure according to the anatomical extent and fracture pattern [12,13]. A sequential approach to reconstruction involving restoration of the medial orbital framework, canthal support, nasal skeleton, and soft-tissue relationships has also been advocated [14]. Accurate primary reconstruction is clinically important because delayed or inadequate treatment may result in persistent telecanthus, epiphora, nasal deformity, facial asymmetry, and other functional or aesthetic sequelae [15]. Furthermore, specific radiographic patterns of injury to the nasolacrimal system have been associated with subsequent epiphora or dacryocystitis, emphasizing the importance of careful radiographic evaluation and postoperative surveillance [16].

The study has several limitations. First, the study included a relatively small consecutive cohort of 20 patients from a single center, and a formal a priori sample size calculation was not performed. Therefore, the precision and generalizability of the findings are limited. Second, the study did not include a comparison group treated using another surgical approach. Consequently, differences in operative time, scar quality, postoperative morbidity, or complications between techniques could not be evaluated. Third, surgical exposure, fracture reduction, and wound healing were assessed using study-specific clinical categories rather than validated quantitative instruments. Observer assessment may therefore have influenced these outcomes.

Fourth, edema and neurosensory status were evaluated clinically rather than using objective quantitative measurements. Fifth, although fractures were classified using the Markowitz and Manson system, outcomes were not stratified according to fracture type because of the small sample size. Two patients required supplementary infraorbital access. Additional surgical approaches may influence operative time, surgical exposure assessment, and postoperative outcomes. Therefore, the observed findings represent outcomes of an H-shaped incision strategy rather than the isolated effect of the H-shaped incision. Finally, follow-up was limited to six months. Longer observation would provide additional information regarding final scar maturation, delayed lacrimal symptoms, stability of reconstruction, and late functional outcomes.

Despite these limitations, the prospective design and serial assessment of postoperative pain, edema, scar quality, neurosensory recovery, and complications provide clinically relevant information regarding the use of the H-shaped approach. Future comparative studies can incorporate larger multicenter cohorts, standardized NOE fracture classification, objective measures of fracture reduction, validated functional assessments, and longer follow-up. Direct comparison with coronal and other limited-access approaches would also permit evaluation of operative time, surgical exposure, scar outcomes, sensory morbidity, and complication rates.

Within the limitations of this study, an H-shaped incision approach provided adequate access for reduction and fixation of selected NOE fractures. The cohort demonstrated satisfactory fracture reduction, progressive resolution of pain and edema, improvement in scar characteristics, and recovery of transient sensory disturbances during six months of follow-up. These findings support the clinical feasibility of the approach while further comparative evidence is required to determine its relative advantages over established techniques.

## Conclusions

In this prospective single-arm study, the H-shaped incision approach provided adequate exposure for reduction and fixation of selected NOE fractures. Fracture reduction was clinically satisfactory in all patients, and postoperative recovery was characterized by progressive reduction in pain and facial edema. Scar scores improved over six months, and no persistent neurosensory deficit or clinically apparent injury to the MCT or nasolacrimal duct was recorded.
